# Dental Management of a Child With Sickle Cell Anemia: A Case Report

**DOI:** 10.7759/cureus.54993

**Published:** 2024-02-26

**Authors:** Sakshi P Kabra, Nilima R Thosar, Neha Pankey

**Affiliations:** 1 Pediatric and Preventive Dentistry, Sharard Pawar Dental College and Hospital, Datta Meghe Institute of Higher Education and Research, Wardha, IND

**Keywords:** sickling, oral consideration, dental management, hemoglobin disease, sickle cell anemia

## Abstract

Sickle cell disease (SCD) has been identified as one of the most prevalent genetic conditions. It alters the shape and function of red blood cells. This brief case report presents a case of a five-year-old male with sickle cell disease who complained of pain in the left mandibular region due to deep proximal caries. Before dental management, a complete fitness evaluation was performed with the help of a pediatrician, followed by informed consent. Dental management includes pulpectomy followed by stainless steel crown placement and Glass ionomer cement (GIC) restoration for superficial caries. Other oral manifestations were observed, including a smooth tongue and mucosal pallor. It was concluded that dentists and health professionals should be knowledgeable of the general and oral anomalies that can be present in individuals with sickle cell anemia in order to take preventive action and implement effective management.

## Introduction

James Herrick was the first to coin the term sickle-shaped red blood cells in 1910 [1]. Vernon Ingram described its pathophysiology in 1957 as the exchange of the glutamic acid, which is the sixth amino acid, a component of the ß chain of Globin (normal adult hemoglobin-HbA), with yet another amino acid, which is valine (hemoglobin-S-HbS), on the 11th chromosome of sickle hemoglobin [2]. Under a lack of oxygen, erythrocytes with a high concentration of hemoglobin S form a sickle shape [3].

This sickling of red blood cells is reversible with elevated oxygen (O2) levels, but the sustained alternative in shape causes the formation of lesions in the cell membrane, resulting in the rigidity of the cells, keeping them from returning to their natural state [4]. Sickle cell disease is diagnosed using a neonatal screening examination that involves drawing blood from the infant's heel to allow hemoglobin electrophoresis to be implanted [5].

The depletion in oxygen-transport capacity causes problems in circulation, including vaso-occlusive conditions, thereby reducing the lifespan of red blood cells from the normal 90-120 days to about 20 [6]. Furthermore, sickled-shaped erythrocytes may obstruct small vessels such as capillaries, arteries, and veins because they bond more closely to the endothelium of cells, forthcoming flow of blood and resulting in a decreased level of oxygen, pain, and necrosis [7].

Oral complications associated with sickle cell disease include mandibular osteomyelitis, mandibular nerve parasthesia, gingival enlargement and glossitis, mucous membrane pallor, and pulpal necrosis [8]. Higher demand for red blood cell formation may result in compensatory expansion of the bones and marrow of the skull and face. It can result in protrusion of the maxilla, with spacing in the upper central incisor teeth, a narrow mandible border, and elevated prominence of the parietal and zygomatic bones [9]. The main reason for the link between sickle cell disease and dental caries appears to be poor hygiene habits [10].

For dental management to be successfully carried out, dental surgeons should understand the physiology along with the pathogenesis of the disease, which will allow them to establish treatment plans while also taking systemic or clinical conditions into account.

## Case presentation

A five-year-old male came with his parents to the department of pediatric and preventive dentistry with the chief complaint of pain in the lower left back tooth region of the jaw for four days. On taking a medical history, his parents revealed that the patient was a known case of sickle cell anemia, and he was under medication for the same.

**Figure 1:**
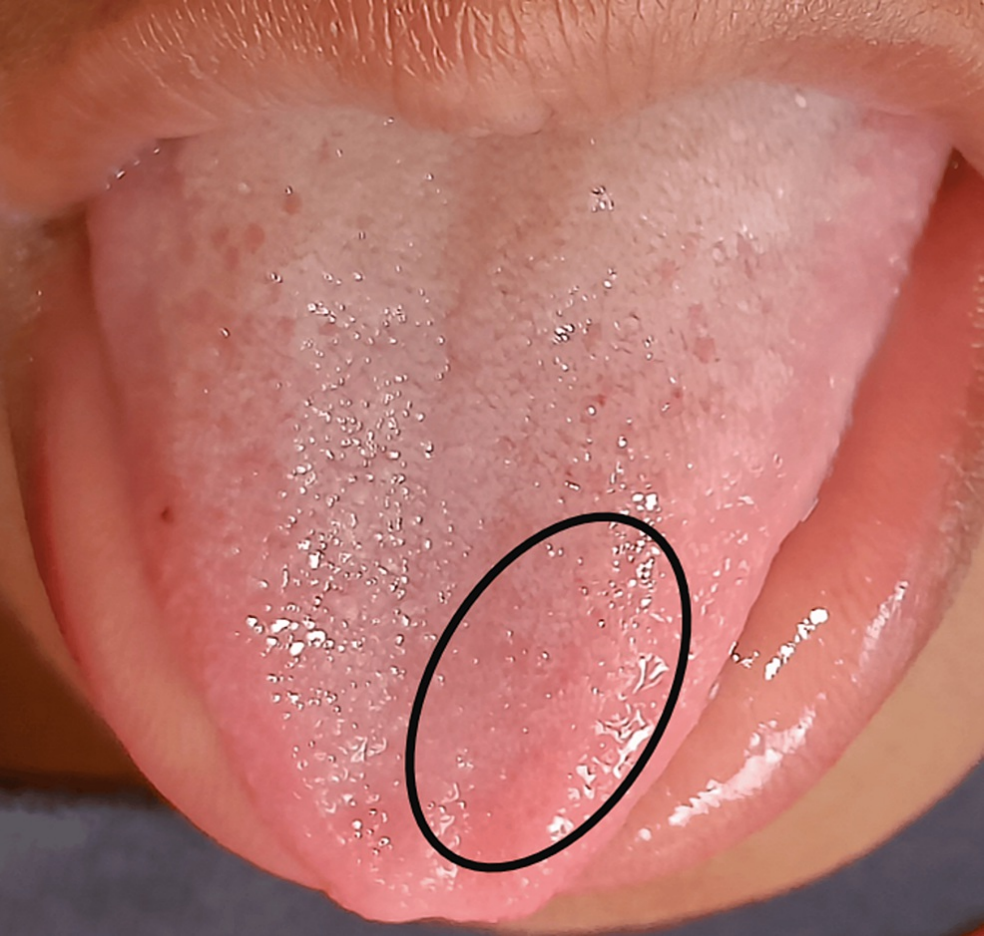
Smooth tongue appearance of sickle cell anemia

Blood investigation revealed sickle cell anemia; the alkaline phosphatase level (222 U/L) was raised. The patient also complained of joint pain, which is a sign of sickle cell anemia. According to the Frankl behavior rating scale, the patient showed definitely positive behavior. The child was conscious, had a normal gait, and was well-oriented to time, place, and person. On intraoral soft tissue examination, slight mucosal pallor and a smooth tongue were noticed (Figure 1). ﻿

**Figure 2:**
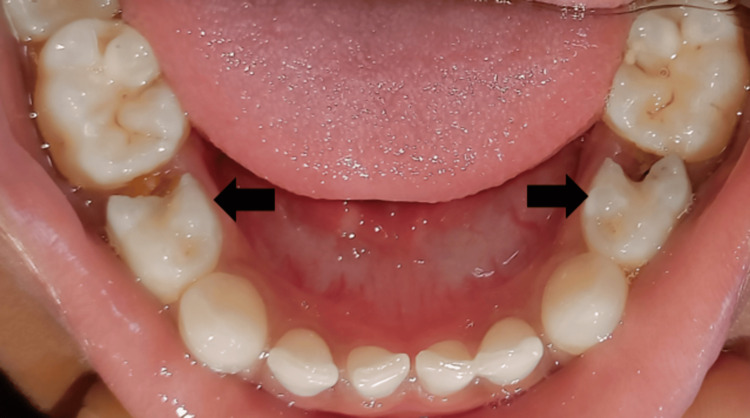
Preoperative image showing disto-proximal caries with 74 and 84

On hard tissue examination, deep proximal caries in relation to 74 and proximal caries with 84 were seen (Figure 2). ﻿

**Figure 3:**
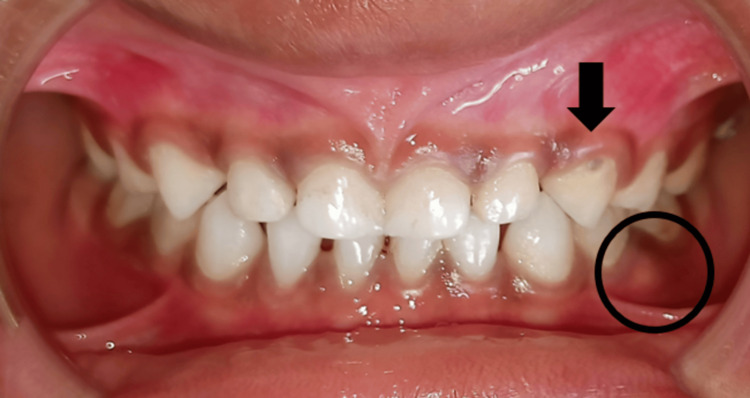
Preoperative image showing smooth surface caries with 63 and dentoalveolar abcess with 74

Smooth surface caries were present with 63 (Figure 3).

**Figure 4:**
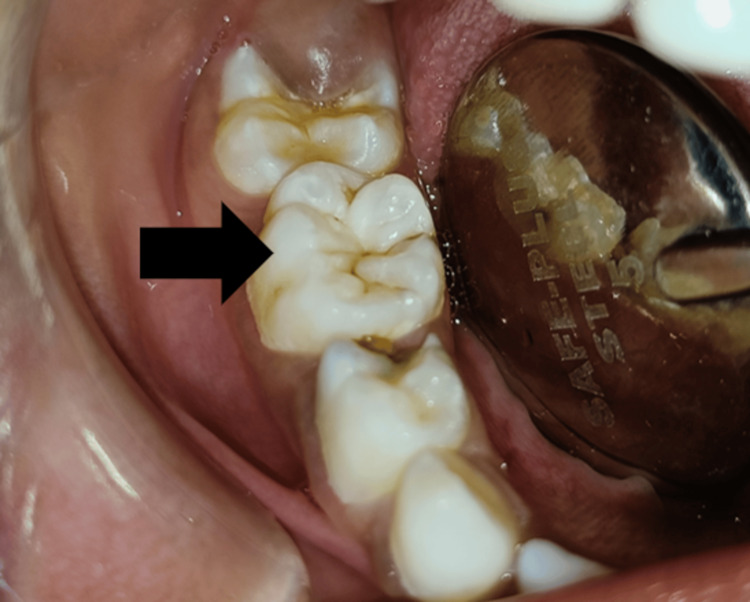
White frosted appearance on the primary second molar with sickle cell anemia.

A white frosted appearance over the primary second molar region was present (Figure 4).

**Figure 5:**
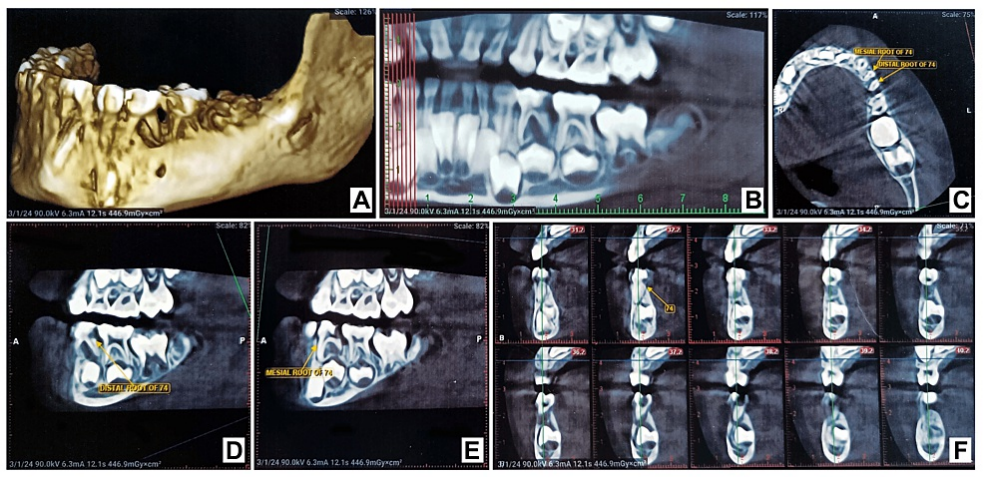
CBCT image showing disto-proximal caries and interradicular bone loss with 74 Sub-images A and B showing disto-proximal caries and interradicular bone loss with 74. Sub-images C, D, E, and F show various aspects of the mesial and distal roots with 74.

Patients had compromised oral hygiene. For bony evaluation, the patient underwent CBCT of the mandibular left quadrant, which depicts deep-disto proximal caries with 74, which involved pulp, and interradicular bone loss were present in the 74 regions. Developing permanent tooth buds were seen (Figure 5).

**Figure 6:**
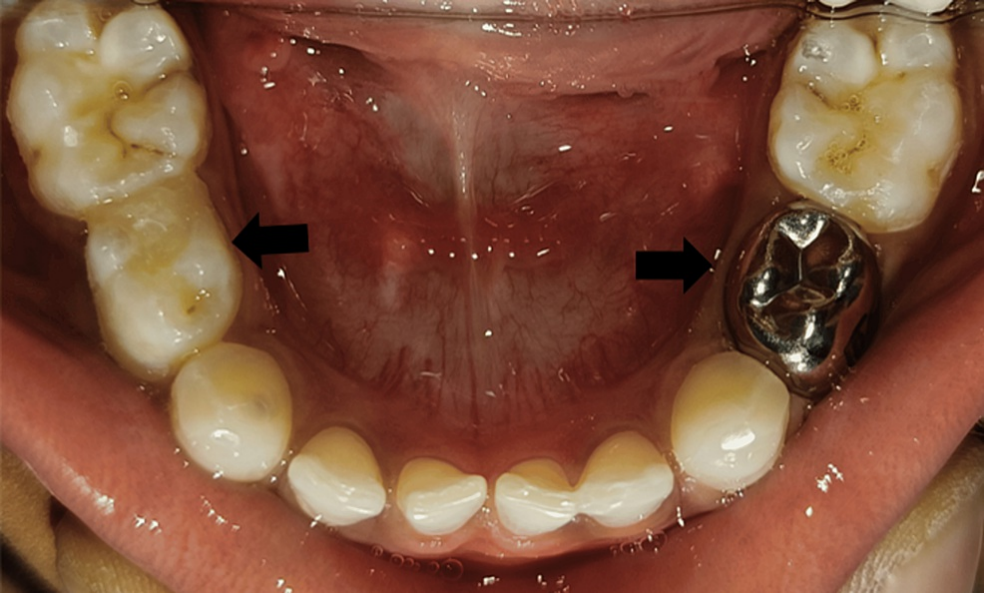
Postoperative image showing stainless steel crown with 74 and composite restoration with 84.

After a physical examination, informed consent, and a fitness certificate taken from the pediatrician, it was decided to go for dental treatment. Before starting the treatment, antibiotic prophylaxis was prescribed for three days. Under aseptic conditions, oral prophylaxis was done; indirect pulp capping using calcium hydroxide was followed by GIC restoration with 84 and 63. Pulpectomy was done in relation to 74, followed by stainless steel crown placement (Figure 6).﻿

Fluoride application and diet counseling were done after one week. After a complete treatment, the patient and his parents were advised to maintain proper oral hygiene, consume a non-cariogenic diet, do tongue cleaning, and apply fluoride four times a year.

## Discussion

Normal adult hemoglobin (Hb-A) molecules consist of four polypeptide chains in which two α units and two β units are present. Each chain contains a heme group, which serves as a platform for the attachment of oxygen molecules. Both chains have distinct amino acid sequences that are drawn together to form unique 3-dimensional structures. The four chains are held together by noncovalent interactions. In SCA, a point mutation converts glutamic acid into valine in the hemoglobin β chain. This type of atypical hemoglobin is known as hemoglobin S (Hb-S), which causes RBCs to harden and change shape. The red blood cell's normal, round disc shape is malformed into a sickle or crescent shape [11].

Sickle cell disease can cause a variety of systemic complications, particularly in areas prone to oxygen deprivation and necrosis [12]. The most common complications are arthralgia, prehepatic jaundice, yellowish discoloration of skin and mucosal pallor, hemorrhagia, sequestration along with infarction of the liver and spleen, impaired pulmonary function, osteomyelitis, and stroke with headaches, seizures, and hemiplegia [13]. In this current case report, the patient described joint pain as a result of insufficient oxygen supply to bones and tissues.

When treating a child with sickle cell disease, the dental practitioner must implement preventive dental approaches such as oral hygiene maintenance, diet control and diet counseling, tooth flossing and tooth brushing instructions, and fluoride applications. Even though oral manifestations are not unique to such a disease, this may indicate the presence of sickle cell anemia [14]. In the present case, the mucosal pallor was mild and concentrated predominantly in the buccal mucosa. The lack of a tongue coating did not exacerbate the tongue's smooth appearance.

In the present case report, hypocalcification of enamel was also observed in the molar region, which leads to a fracture of the tooth. This hypocalcification is seen due to a raised alkaline phosphatase level, i.e., 222 U/L, where the normal range of alkaline phosphatase is between 38 and 126 U/L. Such a type of condition is commonly prevalent in Afro-Caribbean populations and is therefore, mostly seen in Brazil. However, it is rarely seen in other countries [5].

The radiographic examinations, such as cone bean computed tomography (CBCT), were done. It demonstrated a lack of the normal bony trabecular pattern with an elevated radiolucent area due to the reduced number of bony trabeculae in the mandible. Patients with sickle cell anemia generally show a coarse trabecular pattern of “staircase” shape, and secondary development of bony tissue occurs as compensation for bone resorption that occurred during the process of bone marrow expansion, resulting in projections similar to "hair strands" [7]. However, in the present case, no suggestive findings were seen.

These symptoms might alert physicians to the potential that a child has sickle cell disease. The case presented here has particular significance in that the child visited the institute due to severe jaw pain and carious teeth, emphasizing the significance of a thorough clinical evaluation.

## Conclusions

It is essential that all healthcare providers understand the physiology, pathology, and oral signs and symptoms of sickle cell anemia; however, dental practitioners should meticulously obtain the patient's systemic condition through clinical examination along with details about specific features so that any dental treatment can be modified according to the child's needs and limitations. The same as with any management option, the aim should be to maintain oral health and minimize the risk of dental complications due to infections.
